# Quantitative methods for descriptive intersectional analysis with binary health outcomes

**DOI:** 10.1016/j.ssmph.2022.101032

**Published:** 2022-01-22

**Authors:** Mayuri Mahendran, Daniel Lizotte, Greta R. Bauer

**Affiliations:** a Epidemiology and Biostatistics, Schulich School of Medicine & Dentistry, Western University, London, Canada; b Department of Computer Science, Faculty of Science, Western University, London, Canada

**Keywords:** Intersectionality, Health equity, Epidemiological studies, Research design, Biostatistics, CART, classification and regression tree, CTree, conditional inference trees, CHAID, chi-square automatic interaction detector, VIM, variable importance measure, MAIHDA, multilevel analysis of individual heterogeneity and discriminatory accuracy, NHANES, National Health and Nutrition Examination Study, SD, standard deviation, MAD, mean absolute deviation, U.S., United States

## Abstract

Intersectionality recognizes that in the context of sociohistorically shaped structural power relations, an individual's multiple social positions or identities (e.g., gender, ethnicity) can interact to affect health-related outcomes. Despite limited methodological guidance, intersectionality frameworks have increasingly been incorporated into epidemiological studies, both to describe health disparities and to examine their causes. This study aimed to advance methods in intersectional estimation of binary outcomes in descriptive health disparities research through evaluation of 7 potentially intersectional data analysis methods: cross-classification, regression with interactions, multilevel analysis of individual heterogeneity (MAIHDA), and decision trees (CART, CTree, CHAID, random forest). Accuracy of estimated intersection-specific outcome prevalence was evaluated across 192 intersections using simulated data scenarios. For comparison we included a non-intersectional main effects regression. We additionally assessed variable selection performance amongst decision trees. Example analyses using National Health and Nutrition Examination Study data illustrated differences in results between methods. At larger sample sizes, all methods except for CART performed better than non-intersectional main effects regression. In smaller samples, MAIHDA was the most accurate method but showed no advantage over main effects regression, while random forest, cross-classification, and saturated regression were the least accurate, and CTree and CHAID performed moderately well. CART performed poorly for estimation and variable selection. Sensitivity analyses examining the bias-variance tradeoff suggest MAIHDA as the preferred unbiased method for accurate estimation of high-dimensional intersections at smaller sample sizes. Larger sample sizes are more imperative for other methods. Results support the adoption of an intersectional approach to descriptive epidemiology.

## Highlights

• Intersectionality may inform methods for describing health inequity.
• Seven statistical and machine learning methods were evaluated for accuracy.
• For binary health outcomes, method performance was quite sensitive to sample size.
• MAIHDA was preferred for accurate intersection-specific estimation in small samples.
• In larger samples most methods out-performed non-intersectional main effects.

## 1 Introduction

Intersectionality acknowledges that in the context of sociohistorically shaped structural power relations, an individual's multiple social positions or identities (e.g., gender, ethnicity) can interact to affect health-related outcomes (Collins, 2002; Crenshaw, 1989). Since the term intersectionality was first used academically to describe unjust legal processes for Black women (Crenshaw, 1989), this theoretical framework has traversed disciplines and been extended beyond gender and race to other identities or positions that reflect social power structures (e.g., income, age, sexuality, disability) (Bauer et al., 2021; Cho, Crenshaw, & McCall, 2013). Intersectionality can serve as a framework in incorporating social context into epidemiological research, informing conceptualization of research questions, sampling, study design, analysis, and interpretation of results (Agènor, 2020; Bauer, 2014; Bowleg, 2012).

Intercategorical intersectional analyses describe intersectional groups, and differences between them (McCall, 2005). Calculating health outcomes for intersections (defined by a combination of social positions), rather than by combining effects estimated for each position separately, can create more accurate estimates ([Authors' Names Redacted], n.d.). Within descriptive epidemiology, analyses using large population datasets can explore outcomes for high-dimensional intersections (i.e., crossing four or five social positions), including under-studied intersections (Bauer, 2014). We classify as descriptive intersectional analysis methods those able to independently estimate outcomes for co-formed social intersections, by not assuming (as in main-effects regression) that effects of individual social positions are constant across intersections ([Authors’ Names Redacted], n.d.). We use the term “intersectional methods” with the understanding that methods themselves do not make research intersectional. Regression with interaction terms and cross-classification are the most common conventional analysis methods for descriptive intersectional research (Bauer et al., 2021).

Many health outcomes are binary, with a wide range of prevalences, creating challenges when studying high-dimensional intersections. Low-prevalence outcomes are prone to influence by outliers and require large samples to produce suitable numbers of events, problems exacerbated by increasing numbers of intersectional subgroups. For conventional regression analyses with binary outcomes, inclusion of higher-level interaction terms reduces the probability of model convergence and increases the potential for both high variance in estimates leading to wide confidence intervals and for bias away from the null (Greenland, Mansournia, & Altman, 2016; Peduzzi, Concato, Kemper, Holford, & Feinstein, 1996). These issues result in researchers resorting to non-intersectional main effects approaches or limiting the number of intersections under study.

This motivates the evaluation of currently used alternative methods for intersectional analyses of binary outcomes (Bauer et al., 2021). Classification and regression trees (CART), conditional inference trees (CTree), and chi-square automatic interaction detector (CHAID), are data-driven non-parametric methods that apply decision rules to partition data into a single final decision tree, can incorporate any level of interaction, and can identify subgroups for further study or intervention, but do not produce estimates of effect size or variance (Breiman, Friedman, Stone, & Olshen, 1984; Hothorn, Hornik, & Zeileis, 2006; Kass, 1980). Decision trees can also be useful for variable selection, to reduce a list of variables to those most likely to be split on ([Authors’ Names Redacted], n.d.). Random forest aggregates multiple decision trees formed from bootstrapped samples to reduce overfitting (Banerjee, Reynolds, Andersson, & Nallamothu, 2019); it produces no single decision tree or subgroup visualization, but rather a variable importance measure (VIM). Multilevel analysis of individual heterogeneity and discriminatory accuracy (MAIHDA) is a multilevel method which uses individual-level data (Evans, Williams, Onnela, & Subramanian, 2018), with fixed effects for each social position variable and a random intercept for each intersection. MAIHDA uses weighted random intercepts that can reduce overfitting, but appropriateness of fixed and random effects for intersectional interpretation is contested (Evans, Leckie, & Merlo, 2020; Lizotte, Mahendran, Churchill, & Bauer, 2020).

We previously evaluated intersection-specific estimation accuracy of these conventional and alternative methods (except CHAID) for continuous outcomes, using simulated data ([Authors' Names Redacted], n.d.). We found random forest,MAIHDA, and CTree to be more accurate than other methods at smaller sample sizes, while at large sample sizes all methods performed similarly for estimation except for CART, which produced less accurate estimates at both large and small sample sizes ([Authors’ Names Redacted], n.d.). However, method performance and implementation may differ depending on outcome variable type. For example, CHAID analyses require categorical outcomes (Kass, 1980).

The objective of this study was thus to evaluate seven methods (regression with interaction terms, cross-classification, MAIHDA, CART, CTree, random forest, and CHAID) for accuracy in intersection-specific prevalence estimation, alongside a non-intersectional main effects regression and a perfectly specified (but impracticable) regression model, using a variety of simulated but realistic data scenarios. We then sought to demonstrate analyses using National Health and Nutrition Examination Survey (NHANES) 2015 to 2018 data on high blood pressure. Finally, the decision tree methods were assessed for variable selection performance. While epidemiological studies often focus on significance testing and estimation of effect sizes or interactions, accurate outcome estimation for subpopulation groups is an important objective for population health. Therefore, we focus on improving estimation. This study is an opportunity to reexamine how we approach descriptive epidemiology for binary outcomes from both a theoretical and statistical standpoint. While it originated in concerns regarding intersectionality methods, results are relevant to all epidemiological applications that face similar statistical challenges in exploring heterogeneity in binary outcomes.

## 2 Methods

### 2.1 Simulation process

A rare outcome was simulated with an average prevalence of 3% (range: 1.35%–5.60%). A common outcome was simulated with an average prevalence of 15% (range: 7.15%–28.58%). Both outcome types were created with a set of categorical inputs and a set of mixed inputs, resulting in four possible models. Each model was iterated 1000 times for four different sample sizes (N=2000, 5000, 50,000, 200,000). This simulation was structured with identical sample size parameters and input variable combinations as our paper on continuous outcomes ([Authors’ Names Redacted], n.d.), to allow comparison of performance.

Outcome generation formulas were:

#### 2.1.1 Categorical

P(Y=1) = exp (intercept + β1.1 (if X1=1) + β1.2 (if X1 = 2) + β1.3 (if X1 = 3) + β2X2 + β3X3 + β4X4 + β5X5 + β6(if X1=2 & X2=1) + β7(if X1=3 & X2=1) + β8X3*X4*X5).

#### 2.1.2 Mixed

P(Y=1) = exp (intercept + β1X1 + β2X2 + β3X3 + β4X4 + β5X5 + β6 X1*X2 (if X1>1 & X2=1) + β7X3*X4*X5).

Coefficients were sampled so that outcome probability was less than or equal to 1.

The intercept had a value of −3 for the rare outcome, and −1.5 for the common outcome. X1 and X6 were continuous in the mixed inputs model, and categorical (four and three categories respectively) in the categorical inputs model; other variables were binary. Input variable structure is described in Table 1. The input variables resulted in 192 possible intersections of varying sizes. The set of effect sizes for variables X1 to X5 and the interaction terms differed with each iteration. For the rare outcome, effects sizes were selected from a truncated normal distribution (SD=0.30) between 1.24 and 1.80, or 0.20 to 0.76, and for the common outcome between 1.11 and 1.80, or 0.20 to 0.89, on the relative risk scale. X6 was simulated to have no effect on the outcome. Simulation code is provided online (https://github.com/m-mahendran/methods_for_intersectionality_simulation_binary_outcomes). Analyses were conducted in R version 3.6.1 (“R Foundation for Statistical Computing. R: A language and environment for statistical computing,” 2021).

**Table 1:** Description of variables in data generation model input variables.

Variable	Model 1: categorical inputs		Model 2: mixed inputs (categorical and continuous)	
	Type	Distribution	Type	Distribution
X1	Categorical	P(X1 = 0) = 0.25 P(X1 = 1) = 0.25 P(X1 = 2) = 0.25 P(X1 = 3) = 0.25	Continuous (split in quartiles to create intersections for prediction)	mean=0, variance=1
X2	Binary	P(X2=1) = 0.2	Binary	P(X2=1) = 0.2
X3	Binary	P(X3=1) = 0.5	Binary	P(X3=1) = 0.5
X4	Binary	Mediation: P(X4=1 |X3=0) = 0.4 P(X4=1 |X3=1) = 0.7	Binary	Mediation: P(X4=1 |X3=0) = 0.4 P(X4=1 |X3=1) = 0.7
X5	Binary	P(X5=1) = 0.25	Binary	P(X5=1) = 0.25
X6	Categorical	P(X6 = 0) = 0.33 P(X6 = 1) = 0.33 P(X6 = 2) = 0.33	Continuous (split in tertiles to create intersections for prediction)	mean=0, variance=1

Each simulated model resulted in 192 intersections, (4*2*2*2*2*3=192).

### 2.2 Analysis methods

#### 2.2.1 Statistical estimation approaches

For *cross-classification*, the prevalence of the outcome within each intersection was calculated with no further statistical adjustment. *Correctly-specified regression* included only the lower-level coefficients (the intercept and variables X1 to X6) and the interaction terms modeled into the simulated data (X1*X2, X3*X4, X4*X5, X3*X5, and X3*X4*X5). *Saturated regression* included all possible lower-level and interaction terms, and represents a more real-world application wherein the underlying data structure is unknown. Note that interaction terms were for improving overall estimation of intersection-specific outcome prevalence, rather than inference regarding the size or statistical significance of interaction effects. Finally, *main effects regression* was included as a non-intersectional method for comparison. Poisson regression, modified to use robust variance estimation, was used for all single-level regressions, because it produces risk ratio estimates for both rare and common binary outcomes (Zou, 2004). Analyses were run using the R-core function “glm”, and packages “lmtest” and “sandwich” to produce robust variance estimates using the sandwich estimator (Zeileis & Hothorn, 2002; Zeileis, 2006). *MAIHDA models* were run using multilevel logistic regression, using the R-package “lme4” (Bates, Mächler, Bolker, & Walker, 2015). Following MAIHDA modeling practices, fixed effects were assigned to each of the simulated coefficients (X1 to X6), and a random intercept was assigned to each intersection (Evans et al., 2018). While MAIHDA models are typically run using a Bayesian analysis with uninformative priors, we ran a frequentist analysis due to simulation time constraints. Previous simulation has found the main effects estimates to be comparable between the two approaches ([Authors’ Names Redacted], n.d.); other parameters are expected to have similar estimates but have not been exhaustively compared.

#### 2.2.2 Machine learning estimation approaches

*CART*, *CTree*, *CHAID*, and *random forest* models were created with the following R-packages, respectively: “rpart” (Thernau, Atkinson, & Ripley, n.d.), “partykit” (Hothorn et al., 2006), “CHAID” (The FoRt Student Project Team & Hothorn, n.d.), and ‘tuneRanger” (Probst, Wright, & Boulesteix, 2019). CART splitting criterion was based on the Gini rule, with ten-fold cross-validation to select the complexity parameter with minimal cross-validation error. CTree and CHAID models were created using an alpha of 0.05. As CHAID is based on chi-squared analysis, it could only be applied with categorical inputs. Default minimum node size to split for CART, CTree, and CHAID was 20. Random forest models were built with 500 trees, tuned using the parameter mtry by a step factor of 1, and the default minimum final node size was 1. Splitting criterion was based on decreases in node impurity (defined by the Gini index) (Wright & Ziegler, 2017). The VIM was assessed using two measures: impurity-based, which only produces estimates, and permutation-based, which produces estimates and p-values (Altmann, Toloşi, Sander, & Lengauer, 2010).

### 2.3 Study objectives

#### 2.3.1 Primary objective: estimation accuracy

Estimation accuracy was assessed using the mean absolute deviation/mean ratio (MAD). The MAD for each method was calculated as,

$$MAD=\frac{1}{n}\sum_{i=1}^{n}\frac{\left|{\hat{P}}_{i}-P_{i}\right|}{p}\;$$

such that ***n*** was 192 representing 192 possible intersections, ${\hat{P}}_{i}$ was the estimated prevalence of the outcome Y=1 for intersection ***i***, ***P_i_*** was the true prevalence of the outcome Y=1 for intersection ***i***, and ***p*** was the prevalence of the outcome in the entire sample. The true prevalence of the outcome was known by using the outcome generation formula, but not running it through the binary sampling function. Because estimations were for each intersection rather than each individual, accuracy is measured at the subpopulation (intersection) level, rather than the individual level. This emphasizes equal performance across intersections, rather than favouring better performance for more-populated intersections. A MAD of 0 is only achieved by perfect estimation of the prevalence within each intersectional group.

#### 2.3.2 Secondary objective: assessment of variable selection

In population health analyses, even those rooted in theory, there may be more social identity/position variables than can be incorporated into an intersectional analysis. While decision trees do not produce traditional outputs such as variance estimates, they can assist in variable selection to identify relevant variables to include in subsequent analyses that do ([Authors’ Names Redacted], n.d.). Variable selection in this setting is for data analysis planning, not to determine significance or strength of a variable on an outcome. For CART, CTree, and CHAID, variable selection was assessed by the percentage of simulation replicates where each variable (X1 to X6) was used as a splitting variable. Random forest variable selection was assessed by the average impurity-based VIM for each variable, and the percent of iterations that the variable had p<0.05 for the permutation-based VIM. Permutation-based VIM was only assessed for 200 iterations, due to computational time constraints. For our simulated data, decision trees that perform well for variable selection would detect X6 as least relevant.

### 2.4 Sensitivity analysis

We conducted two post-hoc sensitivity analyses assessing the performance of MAIHDA, main effects regression, correctly-specified regression, and cross-classification, at sample sizes 2000 and 5000. First, we simulated four scenarios with a higher outcome prevalence of approximately 50%: categorical inputs, mixed inputs, categorical inputs with larger effect sizes only for interaction effects, mixed inputs with larger effect sizes only for the interaction effects. Second, we assessed the bias and variance of intersection-specific prevalence estimates, using one simulation scenario for the rare, common, and 50% prevalence outcomes with categorical inputs, with one set of effect sizes for each scenario, iterated 1000 times. Bias (the expected difference between modeled estimates and the true parameter value) and variance were estimated for each of the 192 intersections, and results present the median, minimum and maximum values for the 192 intersections. Further description of simulation procedures for sensitivity analyses are provided in Web Appendix 1.

### 2.5 Example NHANES analysis

We used NHANES data to demonstrate and compare differences in results between the methods. Using a multistage probability sample, NHANES is designed to represent the U.S. non-institutionalized population (Chen, Clark, Riddles, Mohadjer, & Fakhouri, 2020). Sixty intersections were formed from sex/gender, race/ethnicity, age, and poverty (income below the US federal poverty line, income above the poverty line). The high blood pressure outcome was defined as systolic blood pressure ≥130 mmHg and/or a diastolic blood pressure ≥80 mmHg (Whelton et al., 2018), each measured by averaging a maximum of three readings. After removal of missing data, final sample size was N=9576. The prevalence of high blood pressure was 41.2%. Analyses included comparing intersection-specific estimations across methods, and comparing final decision tree outputs for variable selection. Other methods-specific outputs are presented in Web Appendix 2.

## 3 Results

### 3.1 Convergence

We considered the feasibility of the different analysis methods for binary outcomes. While other analyses ran smoothly, the saturated regression did not always converge at smaller sample sizes (Table 2). Results for saturated regression models are thus only from converged models.

**Table 2:** Proportion of converged saturated regression models over 1000 iterations by sample size.

	% of models converged			
	N=2000	N=5000	N=50,000	N=200,000
Common binary outcome, categorical inputs	16.7	83.0	100.0	100.0
Common binary outcome, mixed inputs	99.8	100.0	100.0	100.0
Rare binary outcome, categorical inputs	48.0	85.5	100.0	100.0
Rare binary outcome, mixed inputs	98.9	99.8	100.0	100.0

### 3.2 Primary objective: estimation accuracy

**Fig. 1:**
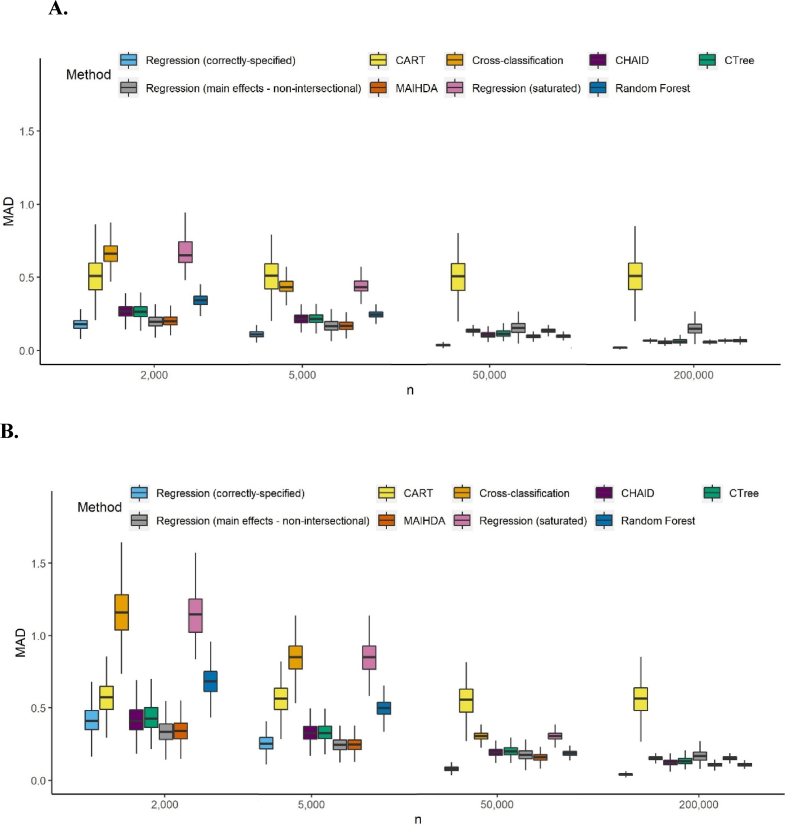

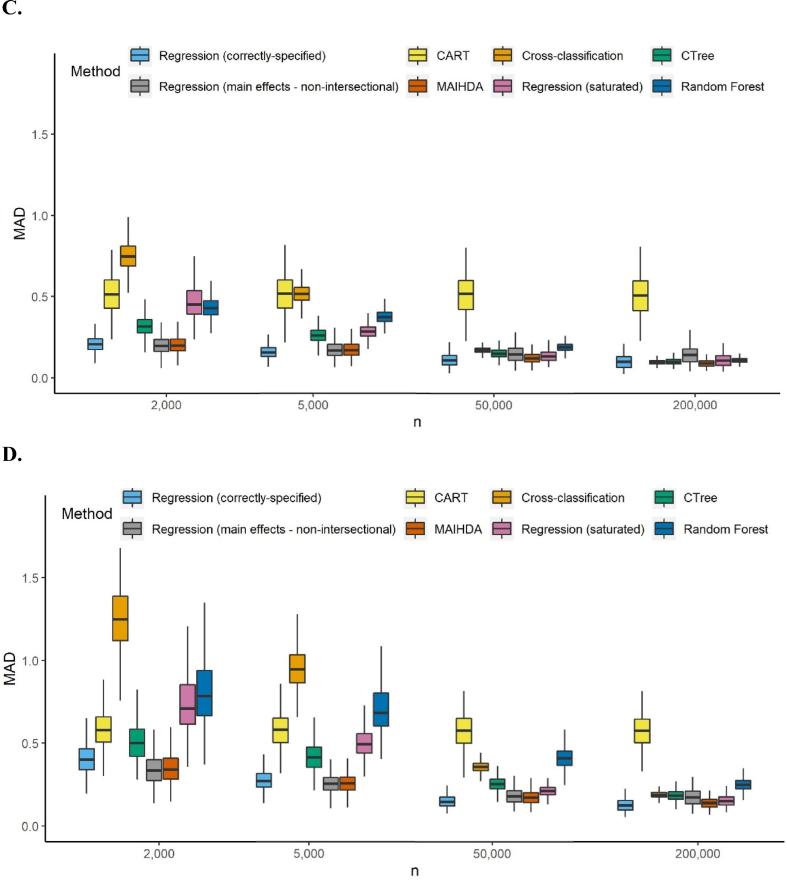
A to 1.D. Boxplots of the mean absolute deviation (MAD) of intersection estimations for four different sample sizes (graph excludes outliers) 1.A. Common outcome with categorical inputs 1.B. Rare outcome with categorical inputs 1.C. Common outcome with mixed inputs 1.D. Rare outcome with mixed inputs. Abbreviations: CART = classification and regression tree; CHAID = chi-square automatic interaction detector; CTree = conditional inference trees; MAIHDA = multilevel analysis of individual heterogeneity and discriminatory accuracy.

Fig. 1 presents the distribution of intersection-specific estimation MAD over 1000 iterations for each of the four scenarios and four sample sizes in the simulated data. In large samples, intersectional methods generally performed well and had higher accuracy than the mis-specified main effect regression. Exceptions were CART, which performed poorly across sample sizes and scenarios, and random forest under just one data scenario. In smaller samples the mis-specified main effects analysis performed better than all other methods except MAIHDA, which performed similarly, and correctly-specified regression for models with common prevalence outcomes. MAIHDA and the implausible correctly-specified regression were the best intersectional methods for all four scenarios at small sample sizes, followed by CTree and CHAID (when applicable).

**Fig. 2:**
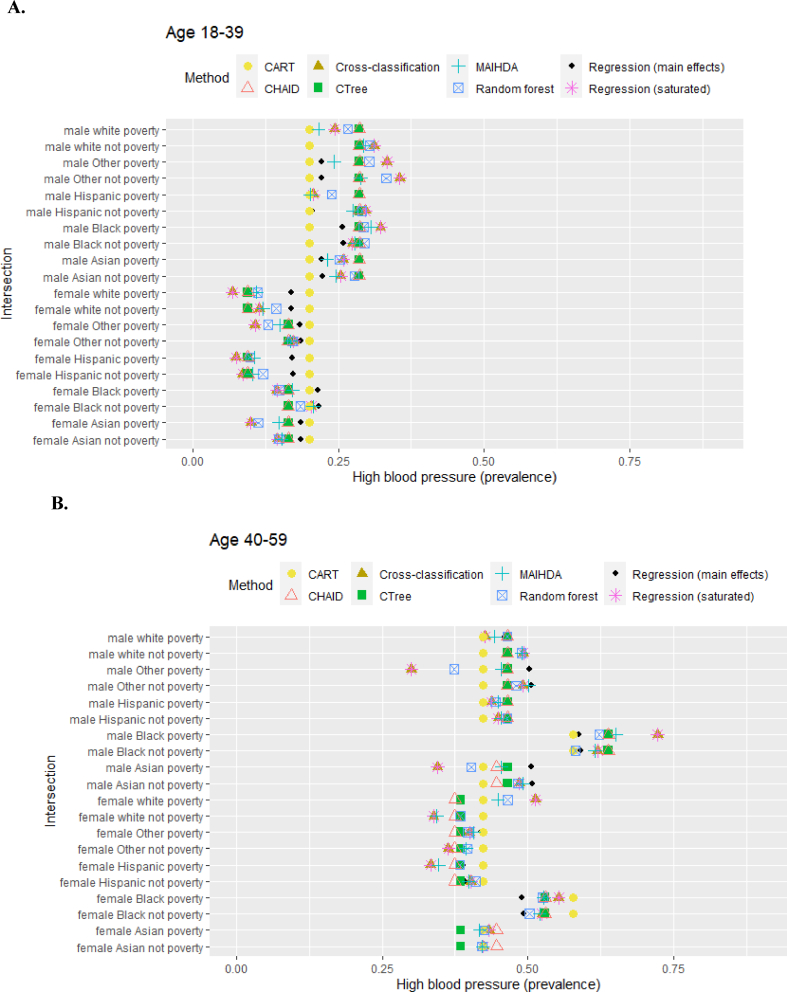

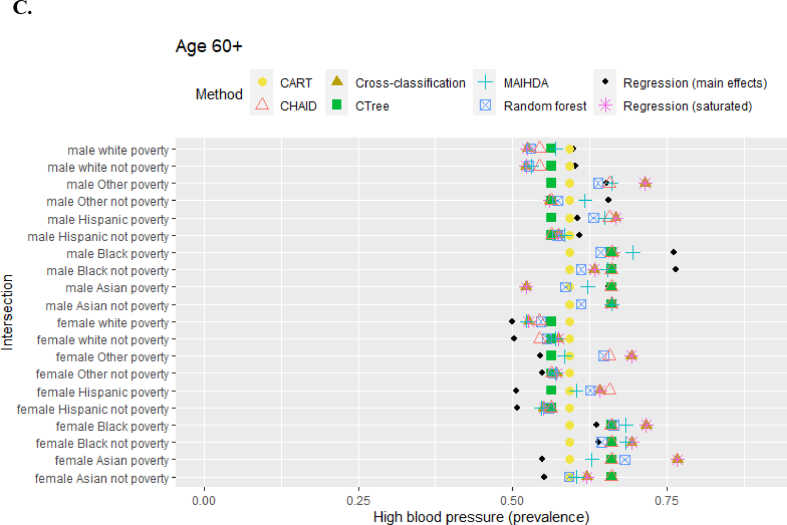
**A to 2.C.** Prevalence of high blood pressure by intersection. Abbreviations: CART = classification and regression tree; CHAID = chi-square automatic interaction detector; CTree = conditional inference trees; MAIHDA = multilevel analysis of individual heterogeneity and discriminatory accuracy.

Fig. 2 presents estimated high blood pressure prevalences for each intersection, by each method, using real-world NHANES data. Choice in methods impacted final estimated prevalences. For example, for white female respondents aged 18 to 39 with poverty-level income, the estimated prevalence varied from 7% to 20%. Comparing the two best-performing methods at smaller samples from the simulation, MAIHDA and main effects, the estimated prevalences were also different. For example, among Black male respondents age 60+ with non-poverty income, main effects estimated 76.5% while MAIHDA estimated 65.3%. For female Hispanic respondents age 60+with poverty-level income, main effects estimated 50.5% while MAIHDA estimated 60.3%.

### 3.3 Secondary objective: variable relevance

Table 3 presents the splitting percentages for the CART, CTree, and CHAID models for the four simulation scenarios. Across all scenarios and sample sizes, CART analysis produced almost no splitting. Therefore, for many iterations estimations were based on only the sample population prevalence, and were equal across all intersections. For CHAID and CTree, splitting on X1 to X5 increased with increasing sample size, and reached 100% in larger samples. X6 splitting frequency was lower than for X1 to X5, but also increased with sample size. The only difference between CTree and CHAID was that the splitting percentages for all variables were slightly higher for CHAID, starting at N=2000. Table 4 presents the average random forest impurity-based VIM's. For models with categorical inputs, X6 was only the least important at larger sample sizes. For the mixed input models where X6 was continuous, X6 was the second most important variable, after X1, even at the largest sample size. Table 5 presents variable selection performance of the permutation-based VIM. A cut off of *P* < 0.05 is effective at maintaining a low detection of false positives in the mixed inputs scenario, where X6 is continuous, but not in the categorical inputs scenario, where X6 is a three-category variable.

**Table 3:** Splitting percentage (% of 1000 iterations) for each variable.

		CART				CTree				CHAID			
		N=2000	N=5000	N=50,000	N=200,000	N=2000	N=5000	N=50,000	N=200,000	N=2000	N=5000	N=50,000	N=200,000
Rare binary outcome, Categorical inputs	x1	0	0	0	0	8	22	99	100	19	37	99	100
	x2	0	0	0	0	5	11	72	99	21	32	90	100
	x3	0	0	0	0	56	78	100	100	73	90	100	100
	x4	0	0	0	0	63	83	100	100	76	92	100	100
	x5	0	0	0	0	27	65	100	100	53	80	100	100
	x6	0	0	0	0	2	4	20	45	12	18	47	67
Rare binary outcome, Mixed inputs	x1	0.1	0	0	0	50	79	100	100	–	–	–	-
	x2	0	0	0	0	10	22	94	100	–	–	–	-
	x3	0.1	0	0	0	49	73	100	100	–	–	–	-
	x4	0.1	0	0	0	52	75	100	100	–	–	–	-
	x5	0	0	0	0	19	47	98	100	–	–	–	-
	x6	0.1	0	0	0	3	5	23	50	–	–	–	–
Common binary outcome, Categorical inputs	x1	0.7	0.4	0	0	51	84	100	100	65	90	100	100
	x2	0.7	0.4	0	0	24	52	98	100	50	76	100	100
	x3	0.5	0.2	0	0	83	95	100	100	92	98	100	100
	x4	0.6	0.2	0	0	86	94	100	100	93	97	100	100
	x5	0.2	0.2	0	0	73	88	100	100	85	95	100	100
	x6	0	0	0	0	7	12	49	77	23	35	68	84
Common binary outcome, Mixed inputs	x1	2	0.4	0	0	83	94	100	100	–	–	–	-
	x2	0.5	0.1	0	0	38	64	99	100	–	–	–	-
	x3	0.6	0.1	0	0	76	90	100	100	–	–	–	-
	x4	1	0.2	0	0	80	91	100	100	–	–	–	-
	x5	0.4	0.1	0	0	62	84	100	100	–	–	–	-
	x6	0.7	0	0	0	6	13	42	64	–	–	–	–

**Table 4:** Random forest average variable importance measure (VIM) (impurity-based: average over 1000 iterations).

	N	X1	X2	X3	X4	X5	X6
Rare binary outcome, Categorical inputs	2000	4	2	2	2	2	3
	5000	4	2	3	3	2	3
	50,000	9	4	19	21	10	4
	200,000	25	10	77	82	39	4
Rare binary outcome, Mixed inputs	2000	33	1	1	1	1	32
	5000	74	3	3	3	2	70
	50,000	376	10	15	16	11	352
	200,000	793	21	54	58	32	712
Common binary outcome, Categorical inputs	2000	18	8	16	17	11	12
	5000	26	12	35	38	21	14
	50,000	124	51	308	338	166	17
	200,000	442	170	1294	1361	645	17
Common binary outcome, Mixed inputs	2000	112	8	12	13	9	101
	5000	226	14	26	27	17	204
	50,000	938	61	211	223	124	782
	200,000	1902	184	822	879	435	1392

**Table 5:** Random forest variable importance measure (VIM) (permutation-based: % of 200 iterations p-value is less than 0.05).

	N	X1	X2	X3	X4	X5	X6
Rare binary outcome, Categorical inputs	2000	19.0	13.5	20.5	25.0	26.5	14.5
	5000	48.0	33.0	73.0	76.5	65.5	16.5
	50,000	100.0	99.0	100.0	100.0	100.0	44.0
	200,000	100.0	100.0	100.0	100.0	100.0	42.0
Rare binary outcome, Mixed inputs	2000	20.5	7.5	8.5	7.5	8.0	2.0
	5000	26.0	11.0	7.0	12.0	14.0	3.5
	50,000	79.0	51.0	55.0	57.0	70.5	0.5
	200,000	100.0	90.0	82.0	81.0	95.5	0.5
Common binary outcome, Categorical inputs	2000	56.5	36.0	69.5	65.0	63.0	7.5
	5000	96.0	81.0	89.5	89.5	85.0	12.0
	50,000	100.0	100.0	100.0	100.0	100.0	46.5
	200,000	100.0	100.0	100.0	100.0	100.0	45.0
Common binary outcome, Mixed inputs	2000	61.5	30.5	55.5	60.0	49.0	1.5
	5000	83.5	59.0	73.0	73.0	74.0	2.5
	50,000	99.5	98.5	91.0	94.0	95.0	1.0
	200,000	100.0	99.5	95.0	96.5	100.0	0.0

Table 6 presents NHANES variable selection results. CART split on fewer variables (i.e., identified fewer as relevant) and resulted in fewer final subgroups than CTree or CHAID. Decision trees visualizations are presented in Web Appendix 2. For random forest models, using the impurity-based measure, age was the most important estimator by a wide margin, followed by gender and race/ethnicity, with income as the least important. Using the permutation-based measure of importance all variables except income were statistically significant at P < 0.05.

**Table 6:** Variable importance measures (VIM) for NHANES high blood pressure.

	CART	CTree	CHAID	Random forest		
	Splitting variable (Yes/No)	Splitting variable (Yes/No)	Splitting variable (Yes/No)	Impurity-based VIM	Permutation-based VIM	Permutation-based VIM P-value
Age	Yes	Yes	Yes	509.927473	0.0552	0.010
Gender	No	Yes	Yes	44.347381	0.005197	0.010
Race	Yes	Yes	Yes	39.163758	0.003238	0.010
Income	No	No	Yes	8.795415	0.000413	0.337

### 3.4 Sensitivity analysis

**Figure 3:**
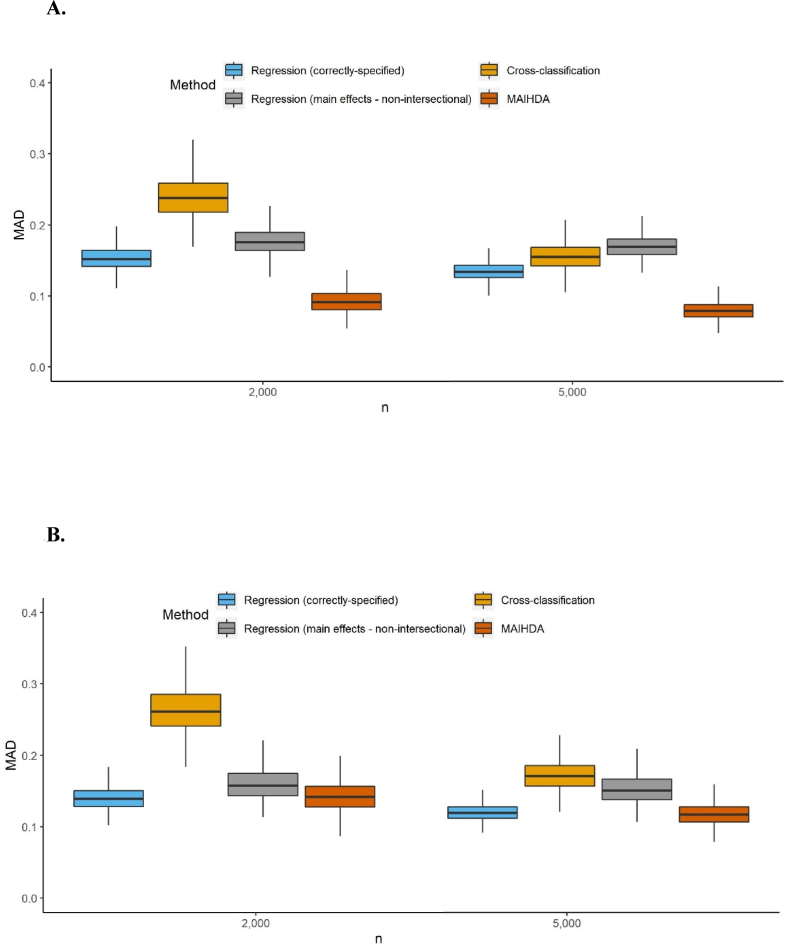

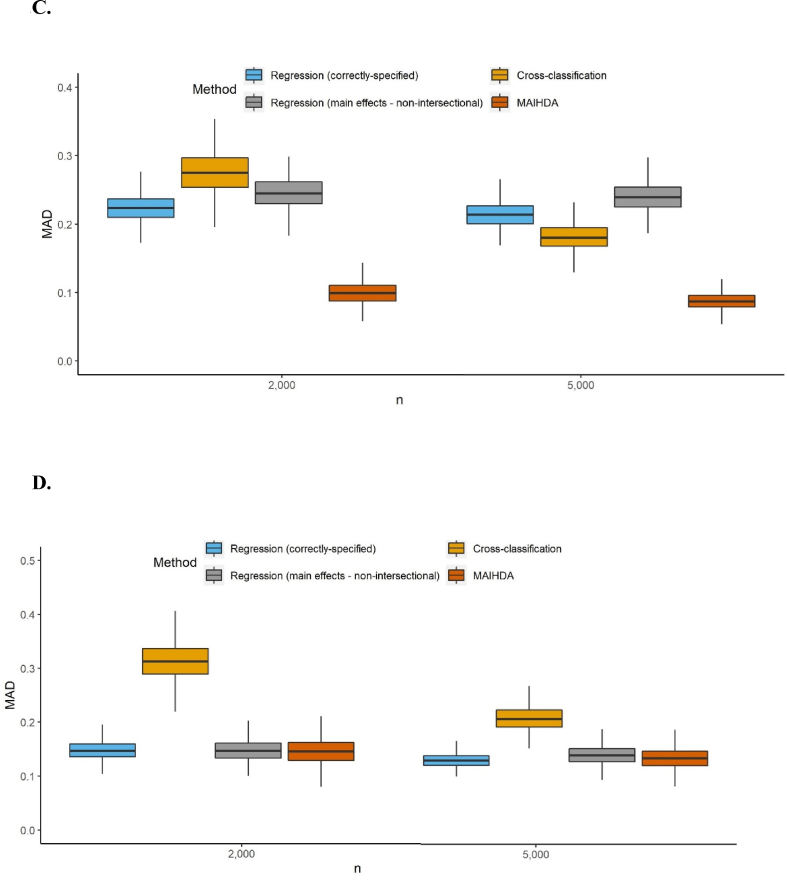
**A to 3.D.** Boxplots of the MAD of intersection-specific estimations for two different small sample sizes, and a simulated outcome prevalence of 50% (graph excludes outliers) A. Categorical inputs B. categorical inputs with larger effect sizes only for interaction effects C. Mixed inputs D. Mixed inputs with larger effect sizes only for the interaction effects. Abbreviations: CART = classification and regression tree; CHAID = chi-square automatic interaction detector; CTree = conditional inference trees; MAIHDA = multilevel analysis of individual heterogeneity and discriminatory accuracy.

Sensitivity analyses present more detailed information on small samples and the performance of main effects analysis. Results from the first set of simulation analyses evaluating method estimation accuracy at 50% outcome prevalence show correctly-specified regression as equivalent or slightly more accurate than main effects regression, and MAIHDA in some scenarios performing better than main effects, correctly-specified regression, and cross-classification (Fig. 3A-D). The second set of analyses evaluating bias and variance of estimates for each intersection (Table 7) shows main effects regression and MAIHDA to generally have smaller variance than the correctly-specified regression or cross-classification, but larger bias. Between main effects regression and MAIHDA, bias and variance of estimates were quite similar, but at the highest outcome prevalence MAIHDA estimates appear more likely to reduce in bias with the increase in sample size between N=2000 and N=5000, while main effects estimates had greater reductions in variance.

**Table 7:** Bias and variance of single simulation models at small sample sizes (median, minimum, and maximum values amongst the 192 intersections).

		Bias		Variance	
		N=2000	N=5000	N=2000	N=5000
Rare outcome prevalence a	Correctly-specified regression	0.02 (−0.1, 0.3)	−0.005 (−0.1, 0.1)	3.06 (0.3, 29.1)	1.09 (0.1, 11.0)
	Cross classification	0.009 (−0.7, 1.1)	−0.002 (−0.5, 0.6)	44.01 (3.1, 362.2)	15.92 (1.2, 165.8)
	MAIHDA	0.04 (−2.8, 2.8)	0.06 (−2.8, 2.7)	1.42 (0.2, 11.7)	0.56 (0.1, 4.1)
	Main effects regression	0.08 (−2.7, 3.0)	0.07 (−2.8, 2.9)	1.39 (0.2, 10.9)	0.54 (0.1, 4.0)
Common outcome prevalence b	Correctly-specified regression	−0.01 (−0.2, 0.4)	−0.03 (−0.3, 0.2)	10.37 (1.2, 93.6)	3.78 (0.5, 36.0)
	Cross classification	0.01 (−2.4, 2.5)	- 0.01 (−2.0, 1.1)	170.72 (13.9, 1053.9)	58.75 (5.3, 591.0)
	MAIHDA	−0.19 (−12.4, 11.1)	−0.26 (−11.7, 10.5)	5.66 (0.5, 30.2)	2.24 (0.2, 14.8)
	Main effects regression	−0.16 (−11.6, 11.8)	−0.20 (−11.4, 11.7)	4.73 (0.5, 40.9)	1.76 (0.2, 14.7)
50% outcome prevalence	Correctly-specified regression	−0.59 (−28.9, 72.6)	−0.47 (−28.7, 73.0)	28.53 (4.4, 121.7)	10.60 (1.6, 49.2)
	Cross classification	−0.03 (−2.4, 2.2)	−0.007 (−1.3, 1.3)	260.6	96.58 (3.5, 1065.0)
	MAIHDA	1.24 (−22.4, 19.7)	0.19 (−12.5, 17.5)	21.94 (0.5, 36.3)	20.98 (0.2, 43.5)
	Main effects regression	2.74 (−36.4, 55.8)	2.74 (−36.2, 56.5)	16.42 (2.6, 74.3)	6.33 (1.0, 28.7)^a^

a Rare outcome prevalence was on average 4%^b^

b Common outcome prevalence was on average 15%.

## 4 Discussion

Challenges in assessing binary outcomes are amplified when assessing outcomes for high-dimensional intersections. At smaller sample sizes our results differed substantially from those in our earlier evaluation of continuous outcomes ([Authors’ Names Redacted], n.d.) with a key difference being that in the binary outcome setting, fewer methods performed well at smaller sample sizes. However, in both the continuous and discrete settings, CART performed poorly.

We modeled 192 simulated intersections, a number typical of published studies using decision tree or MAIHDA methods; researchers studying fewer intersections may want to adjust our “larger” and “smaller” samples accordingly. For example, N=50,000 corresponded to a mean intersection size of N=260, while N=5000 corresponded to 26. Our small-sample analyses intentionally pushed limits for demonstration purposes, and a saturated regression model assessing 192 intersections at N=2000 is not a reasonable expectation.

In larger samples, all intersectional methods but CART were accurate estimators and outperformed non-intersectional main effects. However, at small sample sizes the main effects analysis performed better as an estimator than most methods, except for MAIHDA. Additionally, in smaller samples the traditional methods of cross-classification and saturated regression were poor estimators, and saturated regression was often not feasible given convergence issues.

Our sensitivity analysis assessing the bias and variance of the intersection estimates aimed to explain why at small sample sizes almost all the intersectional methods performed worse than the main effects approach, which given our data scenarios is mis-specified and incompatible with intersectionality. The overall estimation accuracy of a method is attributable to both the bias and variance of the estimates (Geurts, 2009). A main effects analysis's inability to account for variation between intersections was reflected in the bias of the estimates, which was greater for main effects than for a correctly-specified regression or cross-classification. However, at smaller sample sizes the variance of the main effects estimates was much smaller compared to cross-classification or regression with interaction terms, as it estimates fewer effects and is thus less prone to extreme values. The bias-to-variance tradeoff in this situation results in the biased main effects regression often performing equally or better than other methods able to account for intersectionality.

MAIHDA was the only applicable intersectional method (excluding correctly-specified regression as it is not a realistic option) that performed equivalent to or better than a main effects analysis, at smaller and larger sample sizes. Additionally, the two methods were similar in bias-to-variance tradeoff. We suggest that this is because MAIHDA models are comprised of a main effects regression (although main effects are determined differently than for a single-level regression), with additional residual estimates to account for effects within each intersection (Evans et al., 2018). The residuals are weighted so their magnitude is smaller if the intersection sample size is smaller, reducing the impact of the residual on the overall estimate and reducing variance caused by small intersections. For our smallest sample sizes, the residuals are heavily down-weighted, resulting in MAIHDA and main effects regression producing similar estimates. This down-weighting, also called shrinkage, was characterized by Bell, Holman, and Jones (2019) and protects MAIHDA from identifying intersectional effects when evidence (data or signal) is limited.

MAIHDA accuracy surpasses main effects at larger and more plausible sample sizes, with decreased number of intersections, or with a higher outcome prevalence, due to reductions in estimation bias compared to main effects regression. Our NHANES analysis was a real-world example with a high prevalence outcome, assessing a realistic number of intersections given the sample size, and demonstrated the non-equivalence of MAIHDA and main effects regression results. Intersection-specific prevalence estimates differed by up to 10% between MAIHDA and main effects. We recommend MAIHDA for estimating high-dimensional intersectional outcomes, especially when the sample size is small relative to the number of intersections such as in the NHANES example. It theoretically accounts for intersectionality (resulting in reduced bias with increasing sample size) and estimates fewer parameters (resulting in a low variance of estimates even at smaller sample sizes).

Previous studies assessing classification by random forest have concluded that it performs similarly or more accurately than logistic regression, CART, and CHAID (Caruana & Niculescu-Mizil, 2006; Kanerva, Kontto, Erkkola, Nevalainen, & Männistö, 2018; Maroco et al., 2011). However our random forest models produced less accurate intersection-specific estimates, especially in smaller samples. The typical application of random forest, or any decision tree method, may involve hundreds of input variables, and this is when these methods are most advantageous over conventional methods. Even with a large number of intersections, our inclusion of a relatively small number of variables potentially reduced the benefits of a random forest. Additionally, in our data scenarios CART performed poorly. The random forests algorithm used CART to analyze each bootstrapped subsample. Random forests formed using CTree instead may have improved performance (Hothorn et al., 2006).

In both the simulation and NHANES example, CART under-identified relevant variables representing heterogeneities in the data. These results are important given CART's use in intersectionality research as the decision tree method of choice (Cairney et al., 2014; Greene et al., 2019; Villanti et al., 2018). For CTree and CHAID, detection of relevant variables was low at small sample sizes and improved (but also had increasing false positive detection) with increasing sample size. Pruning of CTree or CHAID with the alpha criterion may mitigate Type 1 error issues. For random forest, permutation-based VIM performs well for mixed inputs, as it is less impacted by the bias in the Gini index, which favors splitting on continuous or multicategorical variables (Strobl, Boulesteix, Zeileis, & Hothorn, 2007). The impurity-based VIM better identifies relevant variables for categorical inputs, under conditions of a larger sample sizes and a common outcome. A permutation-based VIM can be used for a set of categorical inputs across outcome frequencies and sample sizes, and relevant variables will be identified as significant more often than the non-relevant variables. However, a permutation-based VIM in these scenarios does run the risk of a high false-positive detection.

To note, variable relevance as referenced in this study is purely quantitative. Our prior study detailed how sequential variable selection by decision tree methods may be a useful tool for variable selection, to narrow down a list of potential variables to be used in other analyses such as MAIHDA or regression with interactions ([Authors’ Names Redacted], n.d.). However, a social position may have real-life impacts and not be detected by data-driven methods. For example, across CTree, CHAID, and random forest, variable relevance was less detectable for rare outcomes, especially at smaller sample sizes. In practice, variable selection for an intersectional approach should consider 1) capabilities of the dataset (for which variables there is enough information) and 2) existing research or community knowledge regarding the possible social structures and powers that would impact the outcome. Tools such as decision trees can provide additional decision support if needed.

Table 8 presents recommendations, as well as quantitative outputs unaddressed by this study. We also present certain methods-specific outputs from the NHANES analysis in Web Appendix 2. We caution careful consideration of the theoretical match to intersectionality of these other outputs. For example, researchers using any method that produces effect estimates must be careful not to focus on significance testing to “prove” intersectionality as a statistical hypothesis, rather than using it as an informative framework (Bowleg, 2012). For decision trees, subgroups formed may be irrelevant to policy or practice if they do not represent reachable real-world groups. Cut-off values and final subgroups are subject to the instability of single decision trees (Li & Belford, 2002), and thus should not be seen as definitive, but rather corroborated with analyses using other datasets (Kreatsoulas & Subramanian, 2018), in addition to existing literature and community knowledge. We strongly recommend presenting measures of variance when possible, both to understand the precision of outcome estimates and to avoid over-emphasis of differences without consideration of within-intersection variation (e.g., via measures of discriminatory accuracy) (Merlo, 2018). The decision tree methods assessed in this study do not inherently produce variance estimates, which indicates an important limitation of these methods.

**Table 8:** Outputs of each method, assessed and not assessed in this study.

	Estimation of binary outcomes	Variable selection	Outputs not assessed in this study
Regression with interactions	Recommended for large sample sizes	Not assessed	Estimation of first-order and interaction effects Conversion of interactions from multiplicative to additive scale for greater public health applicability Variable selection
Cross-classification	Recommended for large sample sizes	Not applicable	Tests of significance between groups (e.g. t-tests) Use of cross-classified groups as categorical variables in regression
MAIHDA	Recommended for all sample sizes	Not assessed	Estimation of main and residual effects How log scale changes interpretation of effect estimates Variable selection Discriminatory accuracy
CART, CTree, or CHAID	CART: Not recommended CTree and CHAID: Recommended for moderate to large sample sizes	CART: Not recommended CTree and CHAID: low power at small sample sizes, high power at high sample sizes, high type 1 error especially with increasing sample size	Comparability of variable splitting to interaction effects identified in traditional regression models
Random forest	Recommended for large sample sizes	Impurity-based: Recommended if all predictors have similar number of categories (e.g. all binary), sample size is large, and outcome is of a common prevalence. Not recommended with mix of continuous and categorical variables. Permutation-based: Recommended strongly if there are at least some continuous predictors. Can be used if variables have a similar number of categories but will result in high type 1 error.	

This study compared multiple methods for describing high-dimensional intersections with binary outcomes, and found MAIHDA most accurate for intersection estimation, especially at smaller sample sizes. We acknowledge that our results may be limited by the simulation data generation process. Additionally, alternative applications of these methods, such as penalized regression with interactions, may improve their performance for estimation or variable selection (Rahman & Sultana, 2017). Future studies may also assess how incorporating survey weights affects the validity of estimation and variable selection. While this study's focus was quantitative performance, we remind researchers that application of intersectionality is more than a methodological choice, but an approach to research process, design, and interpretation. Ultimately, less-conventional methods can allow for better study of high-dimensional intersections, and broaden the possibilities to incorporate intersectional frameworks in epidemiological research, and ultimately to improve health.

## Funding sources

This work was supported by an Ontario Graduate Student Scholarship to MM and by a Canadian Institutes of Health Research Sex and Gender Science Chair to GB [GSB-171372].

## Ethics statement

Research ethics approval was not required for this study, which uses only simulated and publicly downloadable data.

## CRediT authorship contribution statement

**Mayuri Mahendran:** Conceptualization, Methodology, Software, Formal analysis, Data curation, Visualization, Writing – original draft, Writing – review & editing. **Daniel Lizotte:** Conceptualization, Methodology, Writing – review & editing. **Greta R. Bauer:** Conceptualization, Methodology, Writing – review & editing, Funding acquisition.

## Declaration of competing interest

The authors declare no conflicts of interest.

## Footnotes

Appendix A Supplementary data to this article can be found online at https://doi.org/10.1016/j.ssmph.2022.101032.
