# Supplementary material for: Quantitative methods for descriptive intersectional analysis with binary health outcomes
Source: SSM Popul Health. 2022 Jan 22;17:101032. doi: 10.1016/j.ssmph.2022.101032 (PMC8800141; doi:10.1016/j.ssmph.2022.101032)
Supplement: Multimedia component 1 [file mmc1.pdf]

These Supplemental Materials are provided by the authors of the following paper, to provide additional detail for interested readers:

Mahendran, M., Lizotte, D., & Bauer, G.R. (2022). Quantitative methods for descriptive intersectional analyses with binary health outcomes. *SSM – Population Health*.

Web Appendices

Web Appendix 1: Sensitivity analysis procedures 2
Sensitivity analysis 1: 50% prevalence outcome 2
Sensitivity analysis 2: Bias and variance analysis 3
Web Appendix 2: Method-specific results for NHANES analysis 4
2a. Cross-classification results 4
2b. Regression (saturated) results 6
2c. Regression (main effects – non-intersectional) results 8
2d. MAIHDA results 9
2e. CART results 10
2f. CTree results 10
2g. CHAID results 11

Web Appendix 1: Sensitivity analysis procedures

Sensitivity analysis 1: 50% prevalence outcome

A 50% prevalence outcome was simulated with an average prevalence of 50.76% (range: 39.18% to 62.78%). The outcome was simulated with a set of categorical inputs and a set of mixed inputs, and either a mix of small and large effect sizes across the estimates, or small effect sizes for the main effects and large effect sizes only for the interactions. This resulted in four possible models: categorical inputs, categorical inputs with larger effect sizes only for interaction effects, mixed inputs, mixed inputs with larger effect sizes only for the interaction effects. Each model was iterated 1000 times for two different sample sizes (n = 2000, 5000). Outcome generation formulas were:

Categorical

z = intercept + β1.1 (if X1=1) + β1.2 (if X1 = 2) + β1.3 (if X1 = 3) + β2X2 + β3X3 + β4X4 + β5X5 + β6(if X1= 2 & X2=1) + β7(if X1= 3 & X2=1) + β8X3*X4*X5

P(Y=1) = 1/(1+exp(-z))

Mixed

z = intercept + β1X1 + β2X2 + β3X3 + β4X4 + β5X5 + β6 X1*X2 (if X1> 1 & X2=1) + β7X3*X4*X5

P(Y=1) = 1/(1+exp(-z))

This process creates known OR's for each variable, which are the beta-coefficients (βx) exponentiated. The intercept had the following values: -2 for categorical inputs, -1.25 for categorical inputs with larger effect sizes only for interaction effects, -1.25 for mixed inputs, and -1 for mixed inputs with larger effect sizes only for the interaction effects. The structure of the input variables is the same as presented in Web Appendix 1. Different effect sizes for variables X1 to X5 and the interaction terms were selected for each iteration. The effects sizes were selected from a truncated normal distribution (SD=0.30) between 0.20 to 0.89, or 1.11 to 1.80, on the log-odds scale. X6 was simulated to have no effect on the outcome. Simulation code is provided online (https://github.com/m-mahendran/methods_for_intersectionality_simulation_binary_outcomes).

Sensitivity analysis 2: Bias and variance analysis

Bias and variance of intersection prevalence estimates was assessed for the rare, common, and 50% prevalence outcomes with categorical inputs, with 1000 iterations each. Unlike the previous analyses, a fixed set of effect sizes was used for each of the 1000 iterations. Bis and variance were calculated as follows:

$$Bias = \frac{1}{n}\sum_{iter=1}^{n}\left(\hat{P}_{iter} - P\right)$$

$$Variance = \frac{1}{n}\sum_{iter=1}^{n}\left(\hat{P}_{iter} - \bar{P}\right)^2$$

where $\boldsymbol{n}$ was 1000 (the total number of iterations) , $\boldsymbol{\hat{P}_{iter}}$ was the estimated outcome prevalence for the intersection for each iteration (value 0 to 100%), $\boldsymbol{P}$ was the true prevalence of the outcome for the intersection (value 0 to 100%), and $\boldsymbol{\bar{P}}$ was the average estimate of the outcome prevalence for the intersection (value 0 to 100%). The bias and variance was estimated separately for each of the 192 intersections. Results in the manuscript represent the median, minimum, and maximum bias and variance estimates between the 192 intersections. Simulation code is provided online (https://github.com/m-mahendran/methods_for_intersectionality_simulation_binary_outcomes).

Web Appendix 2: Method-specific results for NHANES analysis

2a. Cross-classification results

Web Table 1. Cross-classification results

Intersection	High BP prevalence (%)	n
female Asian 18-39 not poverty	14.6	199
female Asian 18-39 poverty	10.0	30
female Asian 40-59 not poverty	42.4	203
female Asian 40-59 poverty	43.3	30
female Asian 60+ not poverty	62.0	129
female Asian 60+ poverty	76.7	30
female Black 18-39 not poverty	20.3	246
female Black 18-39 poverty	14.5	152
female Black 40-59 not poverty	52.4	296
female Black 40-59 poverty	55.3	76
female Black 60+ not poverty	69.3	241
female Black 60+ poverty	71.6	81
female Hispanic 18-39 not poverty	8.6	337
female Hispanic 18-39 poverty	7.5	161
female Hispanic 40-59 not poverty	40.3	318
female Hispanic 40-59 poverty	33.3	120
female Hispanic 60+ not poverty	55.0	269
female Hispanic 60+ poverty	64.1	131
female other 18-39 not poverty	17.3	75
female other 18-39 poverty	10.7	28
female other 40-59 not poverty	36.4	44
female other 40-59 poverty	40.0	15
female other 60+ not poverty	57.1	35
female other 60+ poverty	69.2	13
female white 18-39 not poverty	11.5	453
female white 18-39 poverty	6.9	102
female white 40-59 not poverty	33.8	399
female white 40-59 poverty	51.3	78
female white 60+ not poverty	57.4	575
female white 60+ poverty	52.5	59
male Asian 18-39 not poverty	25.4	197
male Asian 18-39 poverty	25.8	31
male Asian 40-59 not poverty	48.5	202
male Asian 40-59 poverty	34.5	29
male Asian 60+ not poverty	66.0	106
male Asian 60+ poverty	52.2	23
male Black 18-39 not poverty	27.4	237

male Black 18-39 poverty	32.2	90
male Black 40-59 not poverty	61.9	247
male Black 40-59 poverty	72.2	54
male Black 60+ not poverty	63.2	269
male Black 60+ poverty	66.3	80
male Hispanic 18-39 not poverty	29.7	300
male Hispanic 18-39 poverty	20.7	140
male Hispanic 40-59 not poverty	44.9	245
male Hispanic 40-59 poverty	43.8	80
male Hispanic 60+ not poverty	57.5	268
male Hispanic 60+ poverty	66.7	120
male other 18-39 not poverty	35.5	76
male other 18-39 poverty	33.3	21
male other 40-59 not poverty	49.2	61
male other 40-59 poverty	30.0	20
male other 60+ not poverty	56.0	50
male other 60+ poverty	71.4	7
male white 18-39 not poverty	31.1	440
male white 18-39 poverty	24.4	78
male white 40-59 not poverty	49.2	388
male white 40-59 poverty	42.7	75
male white 60+ not poverty	52.1	654
male white 60+ poverty	52.4	63

2b. Regression (saturated) results

Web Table 2. Saturated regression results

	Estimate (log-odds)	SE	P-value
Intercept	-1.17	0.07	< 0.001
**Gender (ref= Male)**			
Female	-1.00	0.15	< 0.001
**Race/ethnicity (ref=White)**			
Black	-0.13	0.13	0.319
Hispanic	-0.05	0.11	0.671
Asian	-0.20	0.14	0.148
Other	0.13	0.17	0.438
**Income (ref = Above poverty level)**			
Below povert level	-0.25	0.21	0.246
**Age (ref = 18 to 39)**			
40 to 59	0.46	0.09	< 0.001
60 plus	0.52	0.08	< 0.001
**Gender*Race**			
Female*Black	0.70	0.22	0.002
Female*Hispanic	-0.24	0.25	0.333
Female*Asian	0.44	0.26	0.086
Female*Other	0.28	0.33	0.397
**Gender*Income**			
Female*Poverty	-0.27	0.44	0.542
**Gender*Age**			
Female*40 to 59	0.62	0.17	< 0.001
Female*60 plus	1.09	0.16	< 0.001
**Race*Income**			
Black*Poverty	0.41	0.28	0.149
Hispanic*Poverty	-0.11	0.28	0.688
Asian*Poverty	0.26	0.39	0.502
Other*Poverty	0.18	0.40	0.654
**Race*Age**			
Black*40 to 59	0.36	0.15	0.015
Hispanic*40 to 59	-0.04	0.14	0.761
Asian*40 to 59	0.19	0.17	0.255
Other*40 to 59	-0.13	0.22	0.546
Black*60 plus	0.32	0.14	0.023
Hispanic*60 plus	0.15	0.13	0.266
Asian*60 plus	0.44	0.16	0.006

Other*60 plus	-0.06	0.21	0.778
**Income*Age**			
Poverty*40 to 59	0.10	0.26	0.689
Poverty*60 plus	0.25	0.25	0.310
**Gender*Race*Income**			
Female*Black*Poverty	-0.23	0.53	0.664
Female*Hispanic*Poverty	0.48	0.58	0.405
Female*Asian*Poverty	-0.12	0.80	0.876
Female*Other*Poverty	-0.15	0.82	0.857
**Gender*Race*Age**			
Female*Black*40 to 59	-0.49	0.25	0.049
Female*Hispanic*40 to 59	0.51	0.28	0.072
Female*Asian*40 to 59	-0.20	0.29	0.487
Female*Other*40 to 59	-0.21	0.42	0.619
Female*Black*60 plus	-0.70	0.24	0.003
Female*Hispanic*60 plus	0.10	0.26	0.704
Female*Asian*60 plus	-0.60	0.28	0.032
Female*Other*60 plus	-0.36	0.39	0.357
**Gender*Income*Age**			
Female*Poverty*40 to 59	0.83	0.48	0.086
Female*Poverty*60 plus	0.18	0.48	0.712
**Race*Income*Age**			
Black*Poverty*40 to 59	-0.11	0.33	0.739
Hispanic*Poverty*40 to 59	0.23	0.35	0.508
Asian*Poverty*40 to 59	-0.46	0.49	0.351
Other*Poverty*40 to 59	-0.53	0.56	0.345
Black*Poverty*60 plus	-0.36	0.32	0.258
Hispanic*Poverty*60 plus	0.26	0.32	0.422
Asian*Poverty*60 plus	-0.50	0.46	0.276
Other*Poverty*60 plus	0.06	0.50	0.910
**Gender*Race*Income*Age**			
Female*Black*Poverty*40 to 59	-0.43	0.59	0.468
Female*Hispanic*Poverty*40 to 59	-1.21	0.65	0.062
Female*Asian*Poverty*40 to 59	-0.07	0.89	0.937
Female*Other*Poverty*40 to 59	0.18	0.99	0.857
Female*Black*Poverty*60 plus	0.31	0.58	0.590
Female*Hispanic*Poverty*60 plus	-0.39	0.62	0.533
Female*Asian*Poverty*60 plus	0.66	0.85	0.435
Female*Other*Poverty*60 plus	0.19	0.91	0.836

2c. Regression (main effects – non-intersectional) results

Web Table 3. Main effects regression results

	Estimate (log-odds)	SE	P-value
Intercept	-1.59	0.04	< 0.001
**Gender (ref= Male)**			
Female	-0.18	0.02	< 0.001
**Race/ethnicity (ref=White)**			
Black	0.24	0.03	< 0.001
Hispanic	0.01	0.03	0.720
Asian	0.09	0.04	0.019
Other	0.09	0.06	0.159
**Income (ref = Above poverty level)**			
Below poverty level	-0.01	0.03	0.845
**Age (ref = 18 to 39)**			
40 to 59	0.82	0.04	< 0.001
60 plus	1.08	0.04	< 0.001

2d. MAIHDA results

MAIHDA results are presented as the fixed effects and variance estimates. The "null model" only includes random intercepts for the intersections. The "full model" includes all social positions as fixed effects, as well as the intersections as random intercepts. Predictions presented in the main manuscript are based on the "full model". The "null model" variance estimates are presented here to reflect common practice of MAIHDA studies, in order to calculate the Variance Partition Coefficient (VPC) and the Proportional Change in Variance (PCV), as measures of discriminatory accuracy. The formulas for calculating VPC and PCV are as follows, where $\sigma^2_{u(0)}$ is between-stratum variance of the "null model" and $\sigma^2_{u(1)}$ is from the "full model":

$$\text{VPC} = \frac{\sigma^2_u}{\sigma^2_u + 3.29} \text{ x } 100\% \qquad \text{PCV} = \frac{\sigma^2_{u(0)} - \sigma^2_{u(1)}}{\sigma^2_{u(0)}} \text{ x } 100\%$$

Web Table 4. MAIHDA results

**Fixed Effects (Full model)**	**Estimate (log-odds)**	**95% CI[a]**	
Intercept	-1.41	-1.17	- 1.65
**Gender (ref= Male)**			
Female	-0.39	-0.22	-0.57
**Race/ethnicity (ref=White)**			
Black	0.52	0.27	0.77
Hispanic	0.01	-0.24	0.25
Asian	0.17	-0.10	0.46
Other	0.17	-0.17	0.46
**Income (ref = Above poverty level)**			
Below poverty level	-0.02	-0.21	0.18
**Age (ref = 18 to 39)**			
40 to 59	1.28	1.07	1.51
60 plus	1.90	1.70	2.12
**Random Effects (Null model)**	**Estimate**		
Between-Stratum Variance	0.8		
VPC (%)	19.6		
PCV(%)	91.3		
**Random Effects (Full model)**	**Estimate**		
Between-Stratum Variance	0.07		
VPC (%)	2.1		

[a] Confidence intervals computed by bootstrapping

2e. CART results

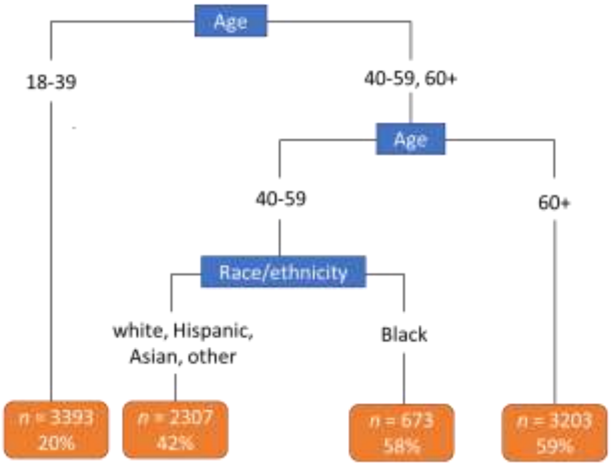

Web Figure 1. CART results

2f. CTree results

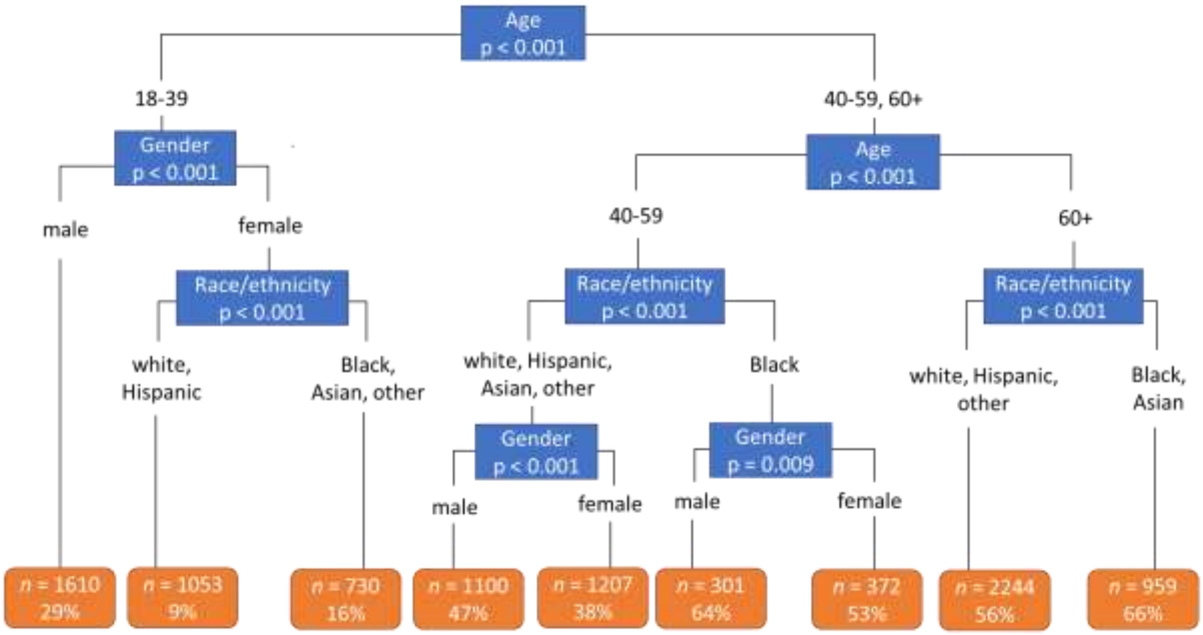

Web Figure 2. CTree results

2g. CHAID results

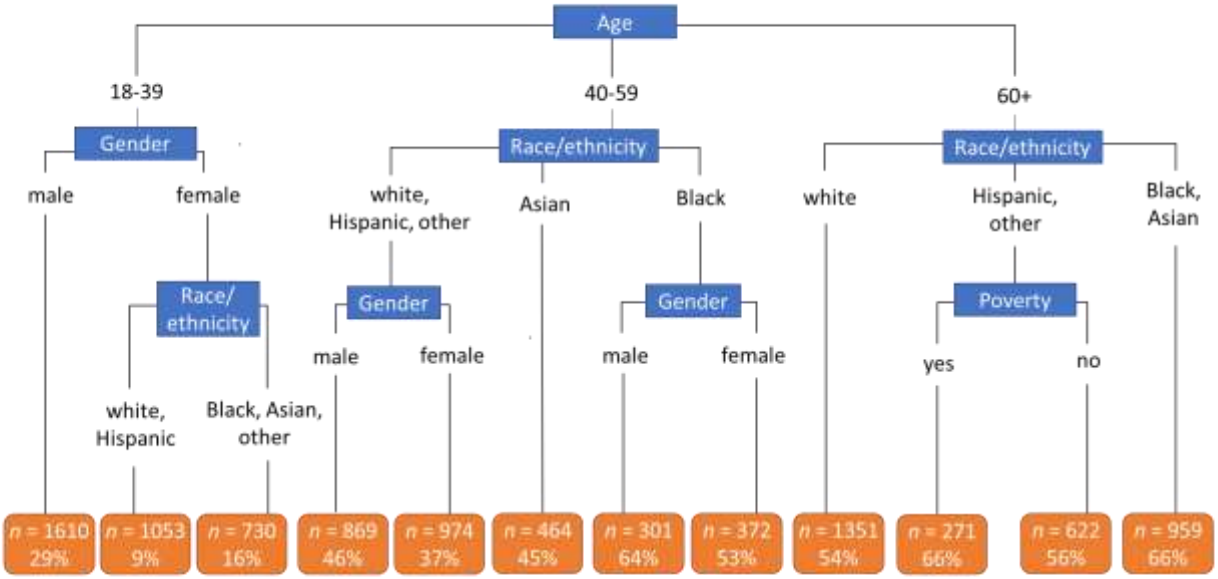

Web Figure 3. CHAID results
